# Continual learning classification method with human-in-the-loop

**DOI:** 10.1016/j.mex.2023.102374

**Published:** 2023-09-14

**Authors:** Jia Liu, Dong Li, Wangweiyi Shan, Shulin Liu

**Affiliations:** a School of Petroleum and Natural Gas Engineering, Changzhou 213164, People's Republic of China; b School of Mechatronic Engineering and Automation, Shanghai University, Shanghai 200072, People's Republic of China

**Keywords:** Artificial immune system, Continual learning, Classification, Human-in-the-loop, H-CLCM: Continual learning classification method with human-in-the-loop

## Abstract

The classification problem is essential to machine learning, often used in fault detection, condition monitoring, and behavior recognition. In recent years, due to the rapid development of incremental learning, reinforcement learning, transfer learning, and continual learning algorithms, the contradiction between the classification model and new data has been alleviated. However, due to the lack of feedback, most classification algorithms take long to search and may deviate from the correct results. Because of this, we propose a continual learning classification method with human-in-the-loop (H—CLCM) based on the artificial immune system. H—CLCM draws lessons from the mechanism that humans can enhance immune response through various intervention technologies and brings humans into the test learning process in a supervisory role. The human experience is integrated into the test phase, and the parameters corresponding to the error identification data are adjusted online. It enables it to converge to an accurate prediction model at the lowest cost and to learn new data categories without retraining the classifier.

• All necessary steps and formulas of H—CLCM are provided.
• H—CLCM adds manual intervention to improve the classification ability of the model.
• H—CLCM can recognize new types of data.

## Graphical abstract

**Figure:**
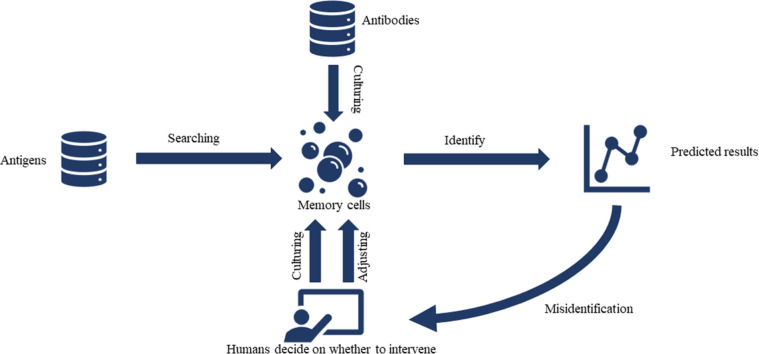

Specifications table

**Table:** 

Subject area:	Computer Science
More specific subject area:	Human-in-the-loop in continual learning
Name of your method:	H-CLCM: Continual learning classification method with human-in-the-loop
Name and reference of original method:	C—CLCM [2] and CLCMNLD [3]
Resource availability:	https://codeocean.com/capsule/8241328/tree/v1

## Method details

### The framework of H—CLCM

**Fig. 1:**
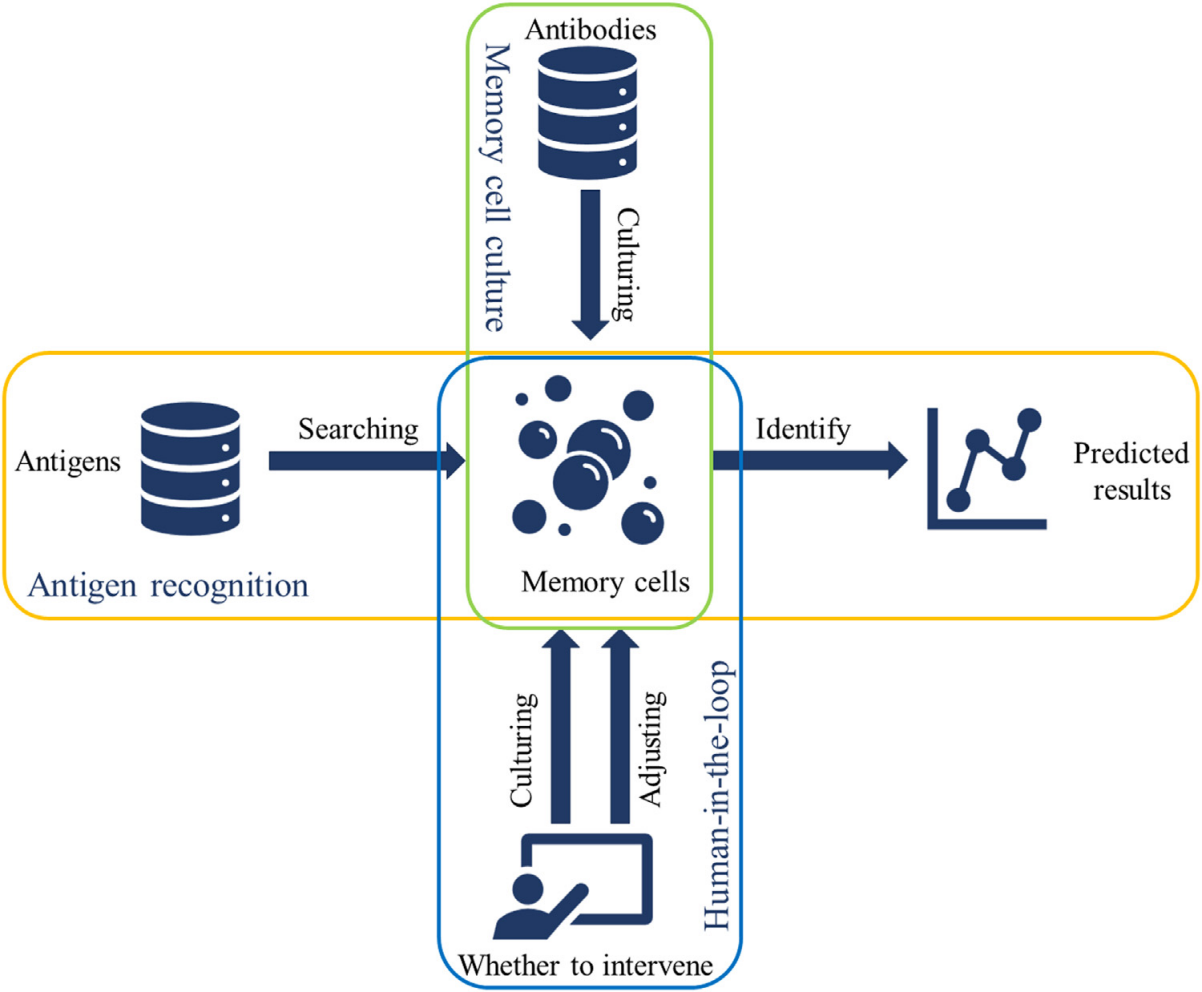
The architecture of H—CLCM.

Fig. 1 illustrates the architecture of the H—CLCM. The framework includes memory cell culture, antigen recognition, and human-in-the-loop process [1]. Among them, memory cells are cultivated by antibodies, which correspond to the training set of traditional machine learning. And the cultivation process of memory cells corresponds to the training process. Antigens refer to the test set, and antigen recognition corresponds to the traditional test process. Although H—CLCM has the same training and testing process as most batch learning methods, batch learning methods need to obtain all available data for model training. When the training is over, the learning is over. However, H—CLCM can learn while training in the process of human-in-the-loop.

### Memory cell culture procedure

**Fig. 2:**
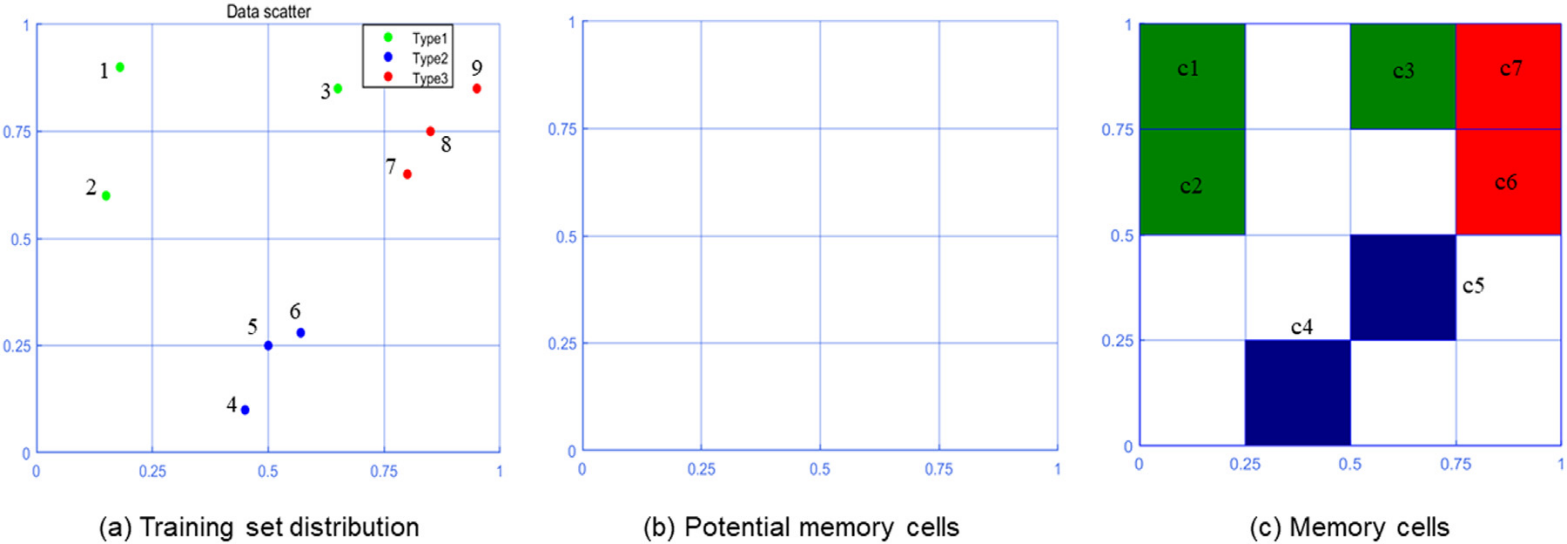
Memory cell culture in two-dimensional space.

Memory cells are cultured through training data (antibodies). Given the passage (*p*) of the memory cell, which divides each dimension into 2*^p^* equal parts, and records the center coordinates of each equal part. For example, when *p* = 2, the center coordinates are [0.125,0.375,0.625,0.875]. The central coordinates of each dimension are combined to obtain the central coordinates of potential memory cells. Find the center coordinate closest to the value of each antibody dimension to locate the corresponding potential memory cells. And the potential memory cells are cultured into memory cells. The culture format is $<c_{1},c_{2},\;...,\;c_{n,\;}p,t_{1},t_{2}\;...,\;t_{m}|c\;\in \;R^{n},p,t\;\in \;N>$. Where $c_{1},c_{2},\cdots ,c_{n}$ refers to the central coordinates of potential memory cells, m represents the passage of memory cells, and $t_{1},t_{2}\cdots ,t_{m}$ represents the number of nuclei of each type stored in memory cells. Fig. 2 illustrates the culture process of memory cells in two-dimensional space. The training set is shown in Table 1, and the parameters of the cultured memory cells are shown in Table 2.

**Table 1:** Training set.

Antibodies	Data
1	(0.18,0.90,1)
2	(0.15,0.60,1)
3	(0.65,0.85,1)
4	(0.45,0.10,2)
5	(0.50,0.25,2)
6	(0.57,0.28,2)
7	(0.80,0.65,3)
8	(0.85,0.75,3)
9	(0.95,0.85,3)

**Table 2:** Initial memory cell parameters.

Memory cells	Parameters
c1	[0.1250,0.8750,2,1,0,0]
c2	[0.1250,0.6250,2,1,0,0]
c3	[0.6250,0.8750,2,1,0,0]
c4	[0.3750,0.1250,2,0,2,0]
c5	[0.6250,0.3750,2,0,1,0]
c6	[0.8750,0.6250,2,0,0,2]
c7	[0.8750,0.8750,2,0,0,2]

### Antigen recognition procedure

**Fig. 3:**
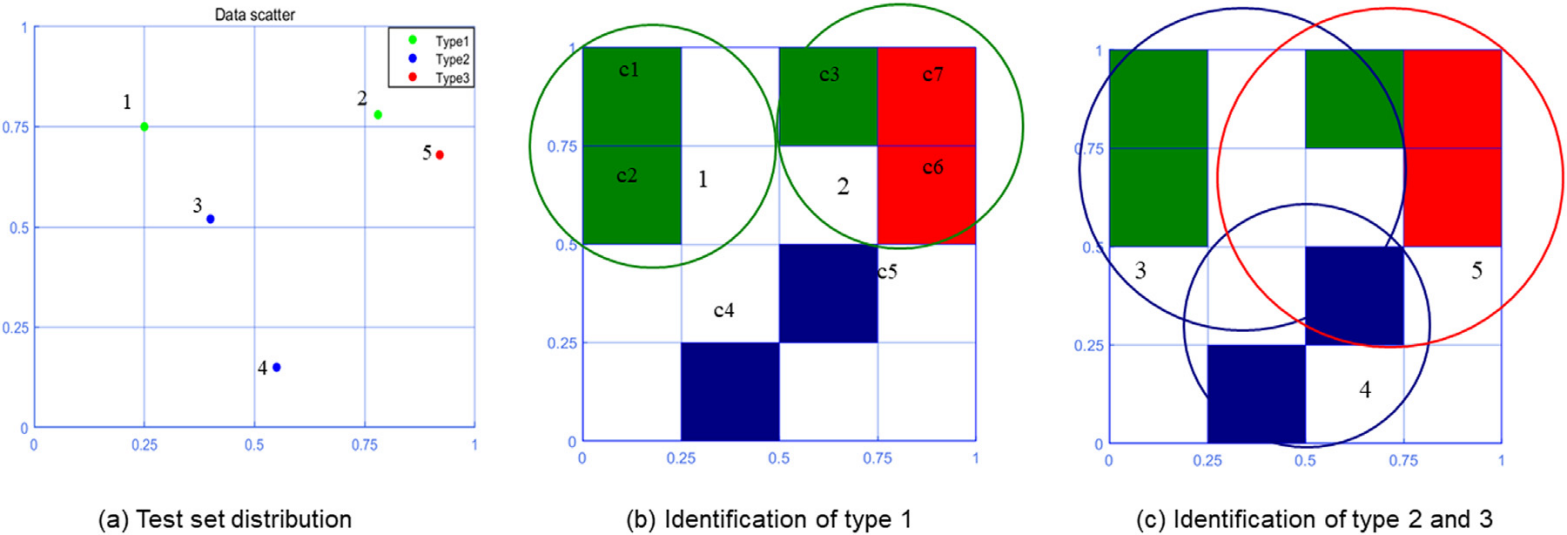
Antigen recognition in two-dimensional space.

Table 3 shows the specific information of the test set. The antigen recognition procedure uses the memory cells generated in Fig. 2(c) for classification. The specific process of antigen recognition is shown in Fig. 3. Table 4 shows the affinity values of various types.

**Table 3:** Test set.

Antigens	Data
1	(0.25,0.75,1)
2	(0.78,0.78,1)
3	(0.40,0.52,2)
4	(0.55,0.15,2)
5	(0.92,0.68,3)

**Table 4:** Affinity.

Antigens	Affinity
1	[0.8886,0.0000,0.0000]
2	[0.6821,0.0000,0.8550]
3	[0.7049,0.4783,0.0000]
4	[0.0000,0.8728,0.0000]
5	[0.3918,0.3389,0.8684]

Use the cultured memory cells to recognize the test data (antigen). According to the data of each dimension of antigen, find out whether there are corresponding memory cells. If the corresponding memory cell exists, search for *k*-1 memory cells adjacent to the memory cell. Combined with the memory cells, a total of *k* memory cells are used to identify the type of antigen. Find the corresponding potential memory cell if the corresponding memory cells are not found. And search *k* memory cells through the potential memory cell to recognize the antigen.

The *k* memory cells adjacent to the antigen are used to calculate the affinity with each type to identify the antigen type. To calculate the affinity, it is necessary to calculate the weight of each type of memory cell. The weight calculation is shown in Eq. (1).

(1) $$x_{i}=\frac{\frac{t_{1i}}{t_{1\max}d_{1}}+\frac{t_{2i}}{t_{2\max}d_{2}}+\cdots +\frac{t_{ki}}{t_{k\max}d_{k}}}{k}\;\left(i=1,2,\cdots ,m\right)$$

where, $d_{l}=E\text{uclidean}\_\text{distance}\left(c_{a},c_{l}\right)\;\left(l=1,2,\cdots ,k\right)$, refers to the Euclidean distance between the antigen and the center coordinate of the memory cell *l*. $c_{a}$ represents the antigen data, and $c_{l}$ represents the center coordinate of the memory cell *l*. $t_{l\max}=\max\left(t_{l1},t_{l2},\cdots t_{lm}\right)\;\left(l=1,2,\cdots ,k\right)$, refers to the largest number of nuclei in the memory cell. $t_{ki}$ represents the number of type *i* nuclei in the *k*th memory cell.

After obtaining the weight of each type (the weight is greater than 0), the weight is normalized to [0, 1) through Eq. (2).

(2) $$f\left(x_{i}\right)\;=\;\frac{2}{\pi }\;\text{arctan}\;\left(x_{i}\right)$$

The recognition of antigen type is determined by Eq. (3):

(3) $$T_{i}=\max\left({aff}_{1},{aff}_{2},\cdots ,{aff}_{m}\right)$$

where, $\left({aff}_{1},{aff}_{2},\cdots ,{aff}_{m}\right)=\left(f\left(x_{1}\right),f\left(x_{2}\right),\cdots ,f\left(x_{m}\right)\right)$, refers to the affinity for each type.

### Human-in-the-loop procedure

The human-in-the-loop process brings human beings into the learning process in a supervisory role [4]. The interaction between humans and data greatly enhances the whole chain of knowledge discovery process and improves the classification ability of the model [5]. In the human-in-the-loop process of H—CLCM, human only participates in decision-making once.

**Fig. 4:**
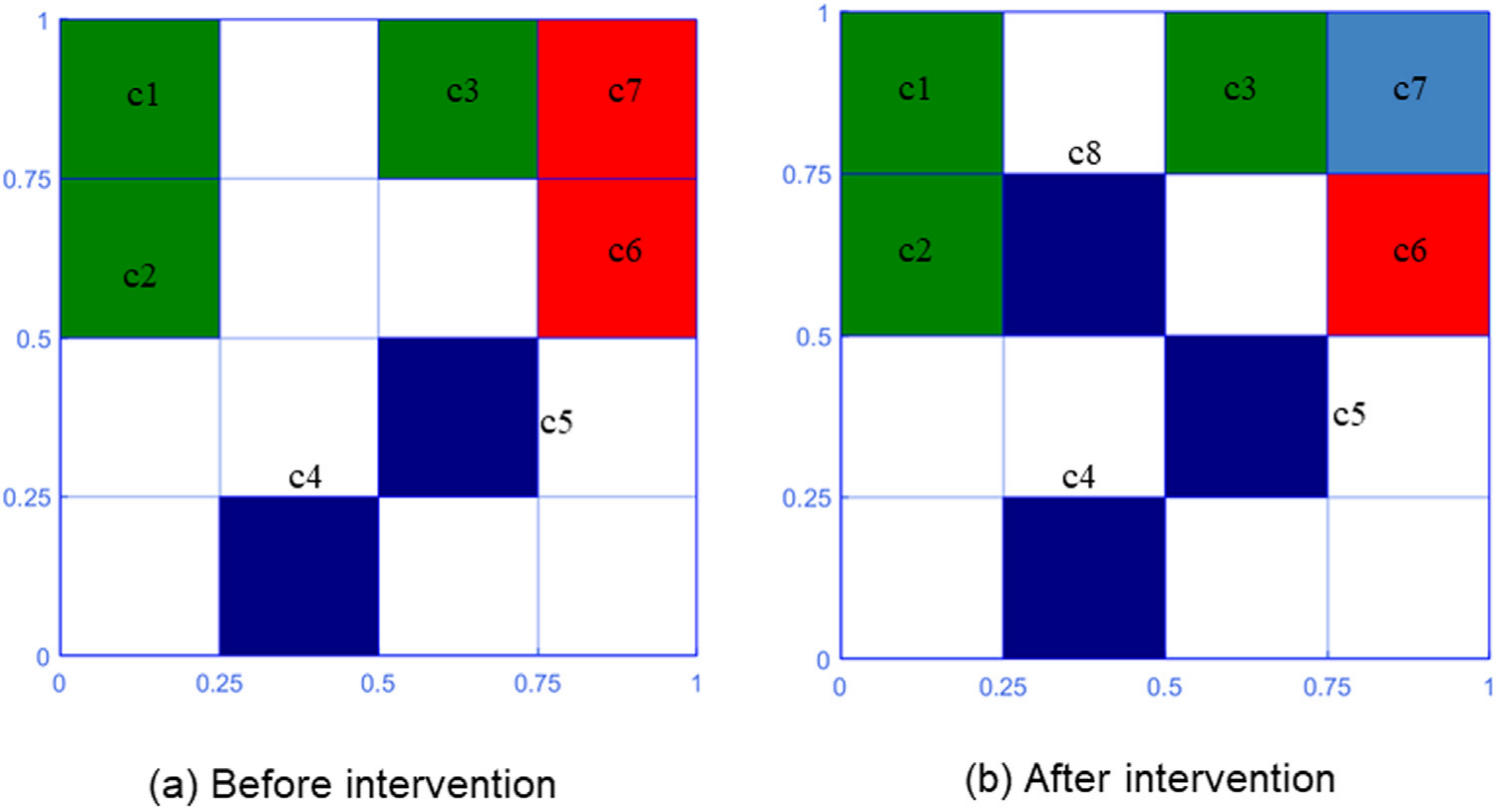
Human-in-the-loop procedure in two-dimensional space.

According to the actual distribution of data and model recognition results, humans decide whether to adjust the memory cells. According to the decision results, the model made two behavioral responses: increasing the number of memory cells and adjusting the type of memory cells. In two-dimensional space, the changes in memory cells after artificial intervention are shown in Fig. 4.

Human-in-the-loop includes the interaction process and actions process. In the interaction process, the model transmits misidentified data to humans. By observing the data distribution in the whole data sample and combining it with the recognition results, humans decide whether to intervene. If no intervention is needed, proceed to the following sample. If intervention is required, perform the actions step.

The actions are as follows:

If the identified wrong sample attacks the potential memory cell, use the memory cell culture process to convert it into a memory cell and store it in the memory cell set. (Generate memory cell, for example, c8 in Fig. 4).

If the wrong sample attacks the memory cell, the number of nuclei in the memory cell structure will be increased by one at the position corresponding to the correct sample type. (Adjust memory cell weights, for instance, c7 in Fig. 4).

At this point, the parameter details of the memory cells are shown in Table 5.

**Table 5:** Changes in parameters of memory cells after intervention (bold Italic for changes).

Memory cells	Parameters
c1	[0.1250,0.8750,2,1,0,0]
c2	[0.1250,0.6250,2,1,0,0]
c3	[0.6250,0.8750,2,1,0,0]
c4	[0.3750,0.1250,2,0,2,0]
c5	[0.6250,0.3750,2,0,1,0]
c6	[0.8750,0.6250,2,0,0,2]
c7	[0.8750,0.8750,2,***1***,0,2]
***c8***	***[0.3750,0.6250,2,0,1,0]***

## Ethics statements

The platform(s)’ data redistribution policies were complied with.

## CRediT authorship contribution statement

**Jia Liu:** Methodology, Software, Validation, Writing – original draft. **Dong Li:** Conceptualization, Methodology, Software, Writing – review & editing. **Wangweiyi Shan:** Investigation, Formal analysis. **Shulin Liu:** Supervision, Funding acquisition.

## Declaration of Competing Interest

The authors declare that they have no known competing financial interests or personal relationships that could have appeared to influence the work reported in this paper.
